# Supplementary material for: Unusual Thermally Induced Blueshift and Emission Amplification of Mn2+ Ions Enable Filter‐Free Luminescent Thermal Imaging
Source: Adv Sci (Weinh). 2026 Jun 1;13(48):e75943. doi: 10.1002/advs.75943 (PMC13336826; doi:10.1002/advs.75943)
Supplement: Supplementary file 1 [file ADVS-13-e75943-s001.docx]

**Supporting Information**

**Unusual Thermally Induced Blueshift and Emission Amplification of Mn^2+^ ions Enable Filter-Free Luminescent Thermal Imaging**

**Y. Abe^1^, M. Szymczak^1,*^, J. Zeler^2^, L. Marciniak^1,*^**

^1^ Institute of Low Temperature and Structure Research, Polish Academy of Sciences,

Okólna 2, 50-422 Wrocław, Poland

^2^

*corresponding author: [l.marciniak@intibs.pl](mailto:l.marciniak@intibs.pl), [m.szymczak@intibs.pl](mailto:m.szymczak@intibs.pl)

*KEYWORDS luminescence thermometry, optical sensors, ratiometric approach, thermal imaging, thermally enhanced Mn^2+^ luminescence*

**Figure S1**. The comparison of XRD patterns for Ca_19_Zn_2_(PO_4_)_14_:Mn^2+^, Ce^3+^ with different concentrations of Ce^3+^ ions.

**Figure S2**. The comparison of XRD patterns for Ca_19_Zn_2_(PO_4_)_14_:Mn^2+^, Ce^3+^ with different concentrations of Mn^2+^ ions.

**Figure S3**. The influence of the Mn^2+^ ions concentration on the *a/b* -a); *c* – b) and *V* – c) in Ca_19_Zn_2_(PO_4_)_14_:Mn^2+^, Ce^3+^ .

**Figure S4**. The emission and excitation spectra of Ca_19_Zn_2_(PO_4_)_14_:0.25%Mn^2+^ measured at 93 K.

**Figure S5**. The comparison of excitation spectra of Ca_19_Zn_2_(PO_4_)_14_:Mn^2+^, Ce^3+^ with different concentrations of Ce^3+^ ions measured at 93 K for Ce^3+^ emission.

**Figure S6**. The comparison of excitation spectra of Ca_19_Zn_2_(PO_4_)_14_:Mn^2+^, Ce^3+^ with different concentrations of Mn^2+^ ions measured at 93 K for Ce^3+^ emission.

**Figure S7**. The comparison of excitation spectra of Ca_19_Zn_2_(PO_4_)_14_:Mn^2+^, Ce^3+^ with different concentrations of Ce^3+^ ions measured at 93 K for Mn^2+^ emission.

**Figure S8**. The comparison of excitation spectra of Ca_19_Zn_2_(PO_4_)_14_:Mn^2+^, Ce^3+^ with different concentrations of Mn^2+^ ions measured at 93 K for Mn^2+^ emission.

**Figure S9**. The comparison of emission spectra of Ca_19_Zn_2_(PO_4_)_14_:Mn^2+^, Ce^3+^ with different concentrations of Ce^3+^ ions measured at 93 K.

**Figure S10**. The comparison of emission spectra of Ca_19_Zn_2_(PO_4_)_14_:Mn^2+^, Ce^3+^ with different concentrations of Mn^2+^ ions measured at 93 K.

**Figure S11**. The emission spectra of Ca_19_Zn_2_(PO_4_)_14_:2%Mn^2+^, 0.5% Ce^3+^ measured as a function of temperature.

**Figure S12**. The emission spectra of Ca_19_Zn_2_(PO_4_)_14_:2%Mn^2+^, 1% Ce^3+^ measured as a function of temperature.

**Figure S13**. The emission spectra of Ca_19_Zn_2_(PO_4_)_14_:2%Mn^2+^, 2% Ce^3+^ measured as a function of temperature.

**Figure S14**. The emission spectra of Ca_19_Zn_2_(PO_4_)_14_:2%Mn^2+^, 5% Ce^3+^ measured as a function of temperature.

**Figure S15**. The emission spectra of Ca_19_Zn_2_(PO_4_)_14_:2%Mn^2+^, 10% Ce^3+^ measured as a function of temperature.

**Figure S16**. The emission spectra of Ca_19_Zn_2_(PO_4_)_14_:0.25%Mn^2+^, 5% Ce^3+^ measured as a function of temperature.

**Figure S17**. The emission spectra of Ca_19_Zn_2_(PO_4_)_14_:0.5%Mn^2+^, 5% Ce^3+^ measured as a function of temperature.

**Figure S18**. The emission spectra of Ca_19_Zn_2_(PO_4_)_14_:1%Mn^2+^, 5% Ce^3+^ measured as a function of temperature.

**Figure S19**. The emission spectra of Ca_19_Zn_2_(PO_4_)_14_:2%Mn^2+^, 5% Ce^3+^ measured as a function of temperature.

**Figure S20**. The emission spectra of Ca_19_Zn_2_(PO_4_)_14_:5%Mn^2+^, 5% Ce^3+^ measured as a function of temperature.

**Figure S21**. The emission spectra of Ca_19_Zn_2_(PO_4_)_14_:10%Mn^2+^, 5% Ce^3+^ measured as a function of temperature.

**Figure S22**. The luminescence decay profiles of Ca_19_Zn_2_(PO_4_)_14_:2%Mn^2+^, 10% Ce^3+^ measured at different emission wavelengths.

**Figure S23**. The excitation spectra of Ca_19_Zn_2_(PO_4_)_14_:2%Mn^2+^, 10% Ce^3+^ measured at different emission wavelengths.

**Figure S24**. The emission spectra of Ca_19_Zn_2_(PO_4_)_14_:10% Ce^3+^ measured as a function of temperature.

**Figure S25**. The emission spectra of Ca_19_Zn_2_(PO_4_)_14_:10%Mn^2+^, 5% Ce^3+^ measured as a function of temperature -a) and normalized spectra – b) upon λ_exc_=365 nm.

**Figure S26**. Emission spectra of Ca_19_Zn_2_(PO_4_)_14_:5% Mn^2+^, Ce^3+^ with different Mn^2+^ ions concentration measured at 83 K– a); the influence of Mn^2+^ ions concentration on the Ce^3+^ to Mn^2+^ emission intensity ratio at 93 K – b); and corresponding CIE1931 chromatic coordinates – c).

**Figure S27**. Thermal dependence of integrated emission intensity of Ce^3+^ ions for different concentration of Mn^2+^ ions – a); thermal dependence of integrated emission intensity of Mn^2+^ ions for different concentration of Mn^2+^ ions – b).

**Figure S28**. Thermal dependence of integrated emission intensity of Mn^2+^ ions for different concentration of Mn^2+^ ions – b); Thermal dependence of *LIR_1_* – a); and corresponding *S_R1_* – b) for different concentration of Mn^2+^ ions.

**Figure S29**. *LIR_1_*, *LIR_2_*, *LIR_3_* for Ca_19_Zn_2_(PO_4_)_14_: 10%Ce^3+^, 2%Mn^2+^ withing heating cooling cycles (temperature changed between 203K and 703 K).

**Table S1**. The traps parameters of Ca_19_Zn_2_(PO_4_)_14_:Mn^2+^, Ce^3+^  and Ca_19_Zn_2_(PO_4_)_14_: Ce^3+^ materials obtained by the deconvolution of TL glove curves using the GlowFit software.

|  | Ce^3+^ | | | Mn^2+^ , Ce^3+^ | | |
| --- | --- | --- | --- | --- | --- | --- |
| No. trap | T_m_/K | E/eV | s/s^-1^ | T_m_/K | E/eV | s/s^-1^ |
| 1 | 310.2 | 0.70 | 1.0E11 | 318 | 0.75 | 3.3E11 |
| 2 | 343.0 | 0.80 | 2.2E11 | 340 | 0.83 | 8.3E11 |
| 3 | 361.0 | 0.85 | 2.8E11 | 358 | 0.88 | 9.7E11 |
| 4 | 378.0 | 0.92 | 6.9E11 | 377 | 0.95 | 1.9E12 |
| 5 | 399.1 | 1.10 | 3.1E13 | 397 | 1.15 | 1.7E14 |
| 6 | 418.2 | 1.30 | 2.0E15 | 416 | 1.28 | 1.4E15 |
| 7 | 437.0 | 1.45 | 2.3E16 | 436 | 1.47 | 4.4E16 |
| 8 | 457.4 | 1.55 | 5.3E16 | 456 | 1.53 | 3.5E16 |
| 9 | - | - | - | 478 | 1.65 | 1.0E17 |

**Figure S30**. The afterglow decay curves for Ca_19_Zn_2_(PO_4_)_14_: 5%Ce^3+^, 2%Mn^2+^ measured as a function of temperature.

**Figure S31**. Thermal evolution of integrated emission intensity calculated in the 560-600 nm spectral range – a) and in the in the 660-720 nm spectral range – b); thermal evolution of *LIR_2_* – c); and *LIR_3_* – d) and corresponding *S_R2_* – e) and *S_R3_* – f) for different concentration of Mn^2+^ ions.

**Figure S32**. Photos of the luminescence of the metal plate covered with the luminescent thermometers captured as a function of time.

**Figure S33**. Intensity maps of the luminescence of the metal plate covered with the luminescent thermometers in Blue channel captured as a function of time.

**Figure S34**. Intensity maps of the luminescence of the metal plate covered with the luminescent thermometers in Red channel captured as a function of time.

**Figure S35**. Intensity maps of the luminescence of the metal plate covered with the luminescent thermometers in Green channel captured as a function of time.

**Figure S36**. The G/B maps of the luminescence of the metal plate covered with the luminescent thermometers captured as a function of time.

**Figure S37**. The G/R maps of the luminescence of the metal plate covered with the luminescent thermometers captured as a function of time.

**Figure S38**. The R/B maps of the luminescence of the metal plate covered with the luminescent thermometers captured as a function of time.

**Figure S39**. Photos of the luminescence of the metal plate covered with the luminescent thermometers captured using IR camera as a function of time.

**Figure S40**. The difference between the real temperature of the plate of Ca_19_Zn_2_(PO_4_)_14_: 5%Ce^3+^, 2%Mn^2+^ heated uniformly to 373 K (*T_real_*) and the temperature measured using G/B ratio (*T_measured_*) calculated in the cross section of the plate.
